# Inhibition of prejunctional parasympathetic pathways by β_3_-adrenoceptor agonists in the isolated pig detrusor: comparison with human detrusor studies

**DOI:** 10.3389/fphar.2023.1177653

**Published:** 2023-05-10

**Authors:** Gianluigi D’Agostino, Stefano Salvatore, Paolo Calvi, Anna Maria Condino

**Affiliations:** ^1^ LMR Unit, University of Pavia, Pavia, Italy; ^2^ Department of Obstetrics and Gynaecology, Vita-Salute San Raffaele University, IRCCS San Raffaele Scientific Institute, Milan, Italy; ^3^ Cellini Clinic, Humanitas Group, Torino, Italy; ^4^ Department of Drug Sciences, University of Pavia, Pavia, Italy

**Keywords:** acetylcholine release, β3-ADR agonist, porcine detrusor, mirabegron, OAB

## Abstract

Adrenergic receptors of the β_3_-subtype (β_3_-ADRs) seem to represent a new target for a more effective pharmacological treatment of overactive bladder (OAB), a wide spread urinary disorder. A promising opportunity for OAB therapy might rely on the development of selective β_3_-ADR agonists, but an appropriate preclinical screening, as well as investigation of their pharmacological mechanism(s), is limited by poor availability of human bladder samples and of translational animal models. In this study, we used the porcine urinary bladder as experimental tool to ascertain the functions of β_3_-ADRs in the control the parasympathetic motor drive. Tritiated acetylcholine ([^3^H]-ACh), mainly originated from neural stores, was released by electrical field stimulation (EFS) in epithelium-deprived detrusor strips from pigs bred without estrogens. EFS produced simultaneously [^3^H]-ACh release and smooth muscle contraction allowing to asses neural (pre-junctional) and myogenic (postjunctional) effects in the same experiment. Isoprenaline and mirabegron produced on the EFS-evoked effects a concentration-dependent inhibition antagonized by L-748,337, a high selective β_3_-ADR antagonist. The analysis of the resultant pharmacodynamic parameters supports the notion that in pig detrusors, as well as in previously described human detrusors, the activation of inhibitory β_3_-ADRs can modulate neural parasympathetic pathways. In such inhibitory control, the involvement of membrane K^+^ channels, mainly of the SK type, seems to play a pivotal role similarly to what previously described in humans. Therefore, the isolated porcine detrusor can provide a suitable experimental tool to study the mechanisms underlying the clinical efficacy of selective β_3_-ADR compounds for human use.

## 1 Introduction

Dysregulation of neural and myogenic pathways is questioned in various forms of lower urinary tract symptoms (LUTS) which produces sensory- or motor-activated incontinence (Andersson, 2010). LUTS are generally divided into storage, voiding and post-micturition components. Overactive bladder (OAB) is a syndrome based on the complaint of urinary symptoms and defined as the presence of urinary urgency, usually accompanied by frequency and nocturia, with or without urgency urinary incontinence, in the absence of urinary tract infection (UTI) or other obvious pathology (Haylen BT et al., 2010). It is a very common condition included in the bladder storage disorders, with multifactorial pathophysiological mechanisms. OAB can result from the alteration of detrusor muscle excitability linked to several myogenic and neurological factors (Andersson, 2010) that urodynamically can determine bladder pressure increase caused by uninhibited detrusor contractions (detrusor overactivity—DO). Muscarinic antagonists are the mainstay of OAB treatment, but their obvious limitations (Apostolidis, 2015) prompted to research novel receptor targets for a more effective treatment of this condition (Andersson, 2015).

Indeed, the focus on sympathetic neurotransmission enriched the current pharmacological portfolio with mirabegron and recently with Vibegron (Patton, 2021), the second β_3_-adrenoceptor (β_3_-ADR) agonist to be approved for the treatment of OAB. Other new putative β_3_-ADR agonists are reported in clinical trials (Andersson, 2017). The clinical success of mirabegron has brought to an increased interest in its mechanism(s) of action. However pharmacological investigations are limited by poor availability of human bladder specimens as well as by the considerable differences in β-ADR expression pattern in different animal species, making extrapolation of findings hard to correlate (Michel and Korstanje, 2016). Therefore, since the pig model is considered, for many aspects, predictive for elucidating integrative bladder physiology (Parsons et al., 2012), we aimed to ascertain the presence and the function of the β_3_-ADR subtype in the porcine detrusor. In particular, the objective of this exploratory study was to demonstrate that β_3_-ADRs are involved in the reduction of parasympathetic excitatory motor drive as described in the human detrusor (D’Agostino et al., 2015). In addition, we explored a possible neural role exerted by Ca^2+ −^activated K^+^ channels (K_Ca_) by means of [^3^H]-ACh release experiments.

For this purpose, we used urinary bladders obtained from male pigs (Cavour strain), bred without estrogens to exclude a possible interference with K_Ca_ channels on detrusor smooth muscle (DSM) (Hanna-Mitchell et al., 2016), and DSM strips prepared urothelium-deprived to minimize the influence of non-neural sources of ACh and ATP (Yoshida et al., 2008). From neural store labeled with tritiated acetylcholine ([^3^H]-ACh), electrical field stimulation (EFS) produced simultaneously DSM contractions and related outflow of radioactivity that were assessed in the presence of β_3_-ADR agonists and antagonists as well as of subtype-preferring compounds for the plasma membrane K^+^ channels, namely BK_Ca_ and SK. The related pharmacodynamics parameters were calculated and compared with published estimates previously obtained in the human detrusor (D'Agostino et al., 2015).

## 2 Materials and methods

### 2.1 Chemicals

The following compounds were purchased: [methyl-^3^H]-choline chloride (2.89 TBq/mmol) from PerkinElmer, Inc. (Boston, MA, United States); Tetrodotoxin (TTX), ω-conotoxin GVIA (ω−CTX), N-[[3-[(2 S)-2-hydroxy-3-[[2-[4 [(phenylsulfonyl)amino] phenyl]ethyl ]amino] propoxy]phenyl] methyl]-acetamide (L748,337), naphtho[1,2-day]thiazol-2-ylamine (SKA-31), (2R,4bS,6aS,12bS,12cR,14aS)-5,6,6a,7,12,12 b,12c, 13, 14, 14a-decahydro-4b-hydroxy-2-(1-hydroxy-1-methylethyl)-12 b, 12c-dimethyl-2H pyran[2″,3'':5′,6']benz [1′,2':6,7] indeno [1,2-b]indol-3 (4 bH)-one (paxilline), 1-(3,5-bis-trifluoromethyl-phenyl)-3-[4-bromo-2-(1H-tetrazol-5-yl)-phenyl]-thiourea (NS11021) and apamine from Tocris Cookson Ltd. (Cabot Park Bristol, UK); hexamethonium bromide, hemicholinium-3, phentolamine hydrochloride and (±)-isoprenaline hydrochloride (INA) from Sigma-RBI (St. Louis, MO, United States); 2-Amino-N-[4-[2-[[(2 R)-2-hydroxy-2-phenylethyl]amino]ethyl]phenyl]-4-thiazoleacetamide (mirabegron) from Santa Cruz Biotechnoloy. Inc. (Santa Cruz, Ca, United States). Mirabegron, L748,337 and K_Ca_ ligands were dissolved in DMSO in stock solutions and appropriate dilutions were prepared daily. The final DMSO concentration in the solutions never exceeded 0.1% vol/vol. At this concentration, DMSO did not influence the EFS-evoked parameters. Other drugs were dissolved in distilled water.

### 2.2 Preparation of porcine detrusor strips

Specimens from the anterior part of the urinary bladder dome of 76 male pigs (Cavour strain, >9 months, carcass weight 160–180 kg), obtained from local abattoir were transported to the laboratory in gassed (95% O_2_ and 5% CO_2_) Krebs’ solution at 6°C containing (mM): NaCl 120, KCl 4.7, MgSO_4_ 0.6, NaHCO_3_ 25, KH_2_PO_4_1.2, CaCl_2_ 2.0 and glucose 10 (pH 7). DSM strips (20 mm long, 4 mm wide), prepared by removing urothelium with the edge of a scalpel. Urothelium removal was confirmed by histological examination. Four strips were mounted isometrically under an initial tension of 2 g in 2 ml chambers superfused with Krebs’ solution at 37°C. Electrical field stimulation (EFS) was applied by means of two platinum electrodes placed parallel to the preparation, which activate nerves, similar to triggering bladder contractions *in vivo*.

The study was approved by the Animal Ethics Committee of University of Pavia and complies with the current European laws in adherence to guiding principles of Three Rs (3R s) for more ethical use of animals.

### 2.3 Labelling and release experiments

After a 30 min equilibration period, neuronal release of [^3^H]-ACh was assessed according to the procedure previously described for the human bladder (D'Agostino et al., 2015). Briefly, the preparation was incubated for 45 min with [methyl-^3^H] choline (92 kBq/ml) to label neuronal ACh stores under EFS applied by 10 s pulse trains delivered at 10 Hz (0.2 m duration, 60 V/cm, 60 s apart). Following loading, the preparations were washed out for 120 min by superfusion at a constant rate of 2 ml/min (Minipulse 2HP8 flow inducer, Gilson Medical Electronics, Middleton, WI, United States). Hemicholinium-3 (10 μM) and phentolamine (1 μM) were present in the superfusion solution throughout the experiment to prevent choline uptake and influence of α-ADRs, respectively. Starting at the 121th min (zero time), the fluid was collected continuously in 3 min periods (6 ml samples) and aliquots (1 ml) were measured in 3 ml of Ultima Gold scintillation cocktail (Packard BioScience, Groningen, Netherlands) by liquid scintillation spectrometry (Tri-Carb 2700TR, PerkinElmer, Shelton, CT, United States). Quench correction curves were established and external standardization was used for counting efficiency. Tritium content was expressed in disintegration per s (Becquerel) for gram of dry weight of the tissue (Bq/g) determined at the end of the experiment.

#### 2.3.1 Pre- and postjunctional experimental protocols

EFS at 20 Hz evoked simultaneously submaximal DSM contraction and [^3^H]-outflow, which can be regarded as a reliable indicator of [^3^H]-ACh release from postganglionic cholinergic nerve terminals (D’Agostino et al., 2000). EFS was delivered in individual preparation with different parameters in two protocols (A and B).

Protocol A. DSM strips were stimulated by intermittent EFS (IS) delivered with six trains of 9 s at 20 Hz (0.1 m durations, 90 V/cm, 33 s apart) according to D’Agostino et al. (2015). Two EFS (S_1_ and S_2_ evoked at ninth min and 54th min after zero time, respectively) evoked an increase of ^3^H]-outflow that was calculated as difference between the total tritium outflow during 3 min stimulation plus the following 12 min (evoked-outflow period) and the calculated spontaneous outflow. The decline for the spontaneous outflow was calculated by fitting a linear regression line to the values (expressed in Bq/g) of 3 min-samples before and after the evoked-outflow period. In control situation (S_2_/S_1_ in the absence of tested drugs), the ratio between the [^3^H]-outflows evoked during the two stimulation periods (S_2_/S_1_) was calculated and considered as 100% reference value. The change in this ratio caused by the tested drugs was taken as a measure of their percentage effect at neural side.

DSM contractions (IC_1_ and IC_2_) evoked by S_1_ and S_2_, respectively, were measured as the mean value of six contractions. This value was considered as 100% reference in control situation (IC_2_/IC_1_ in the absence of tested drugs). Any variation in IC_2_/IC_1_ ratio caused by drug exposure compared to the equivalent ratio in control experiments was taken as a measure of the muscular effect. The drugs were added 8 min (agonists) or 30 min (antagonists and toxins) before the onset of S_2_. Accordingly, concentration-response curves (CRCs) for agonists were constructed in the absence and in the presence of antagonists.

Protocol B. After the washout period (zero time), spontaneous [^3^H]-outflow was measured for 12 min and thereafter DSM strips were stimulated by continuous EFS (CS) delivered with 3 s trains at 20 Hz (0.75 m durations, 20V/cm, 60 s apart) according to Soder et al. (2013). When DSM twitch contractions were reproducible, related EFS-evoked [^3^H]-outflows were measured and those evoked during two 15 min periods (P1 and P2, starting at 26th min and 75th min after zero time, respectively) were compared. The respective amount of radioactivity was calculated as area under curve (AUC) that is the value resulting from the difference between the total tritium outflow during 15 min stimulation and the calculated spontaneous outflow. The decline for the spontaneous outflow was calculated as described in protocol A. Drugs (K_Ca_ activators and blockers) were exposed 15 min before P2 and the resultant P2/P1 calculated. The change of this ratio compared to that observed in control situation (P2/P1 in the absence of tested drugs) was taken as a measure of the percentage effect caused by K_Ca_ ligands.

DSM contractions (CC_1_ and CC_2_) were assessed as the mean value of the 15 contractions evoked during P_1_ and P_2_, respectively. Any variation in CC_2_/CC_1_ ratio caused by drug compared to the equivalent ratio in control experiments was taken as a measure of the muscular effect.

### 2.4 Data analysis

The present study and analyses were designed to be exploratory, but not to test a pre-specified statistical null hypothesis. Therefore, *p* values reported here should be considered as descriptive and not as hypothesis testing.

The amounts of ACh release were measured as S_2_/S_1_ and P2/P1 ratios by Peak (software program from University of Pavia). The effectiveness of ACh release in producing isometric DSM contraction was assessed on PowerLab apparatus (ADInstruments, Castle Hill, Australia) by mean of LabChart 3.6 software.

Results are expressed as mean ± SD, with n indicating the number of preparations used for a particular set of experiments: no outliers were excluded from data analysis and presentation and the *n* number was always equal or higher than five. Only one experiment was performed per preparation.

Drug potency estimates were evaluated for agonists as -log EC_50_ (negative log of the molar concentration-producing half-maximal effect) by non-linear curve fitting (best-fit value Delta log EC_50_, GraphPad Prism, version five; GraphPad Software Inc., San Diego, CA). Efficacy was expressed as Emax (maximal effect vs control). Apparent affinity (p*K*_i_) estimates were calculated for antagonists (D'Agostino et al., 2015). One sample *t*-test using 100% as reference value was applied to evaluate the effects caused by drugs. *p* < 0.05 values were considered statistically significant.

## 3 Results

### 3.1 Neural and muscular effects evoked by intermittent EFS in pig DSM strips

**FIGURE 1:**
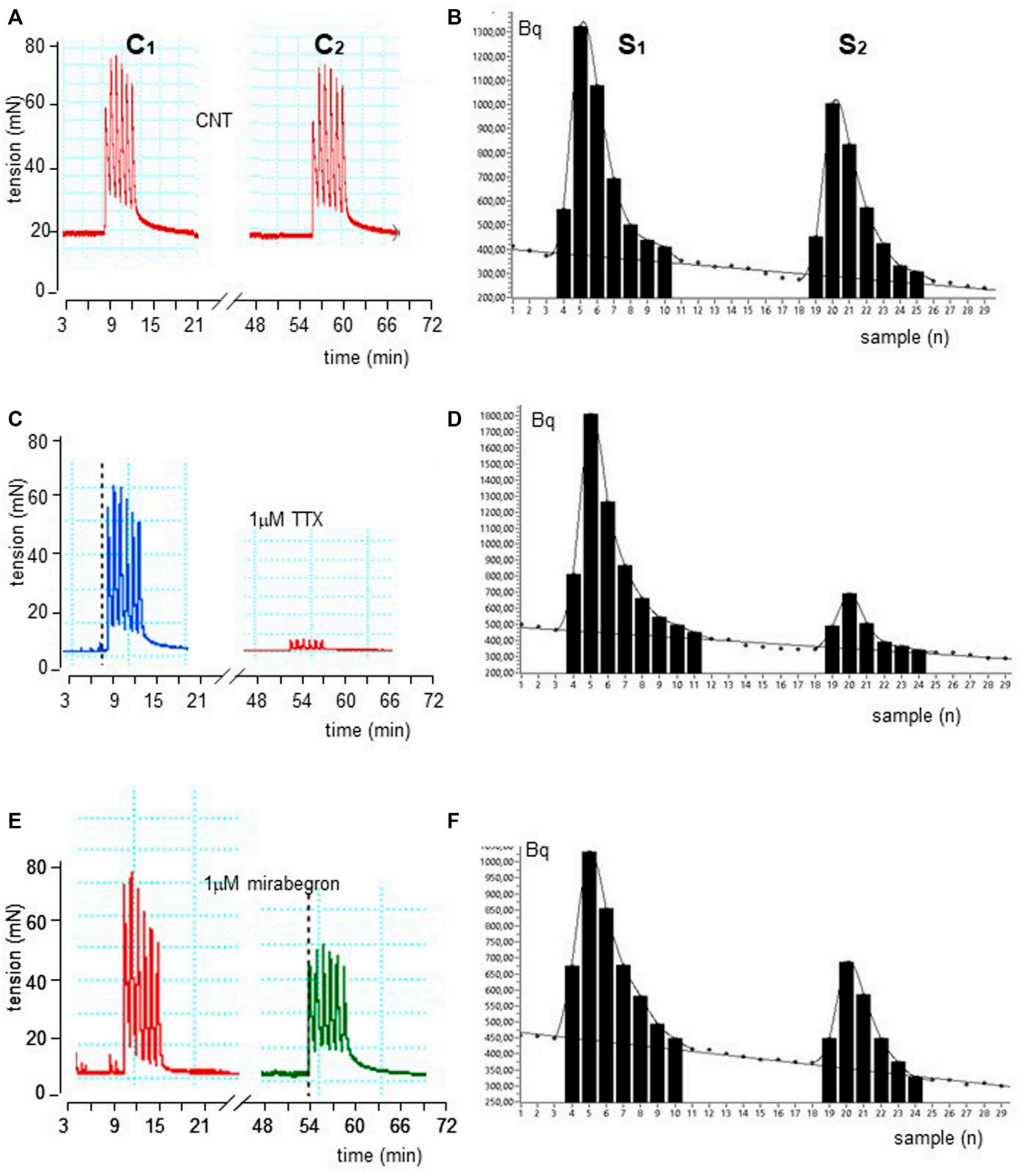
Time course illustrated by original drawings of experiments in pig DSM strips according to protocol A (see Methods for details). Intermittent EFS (IS) delivered at 20 Hz 33 s apart caused simultaneously contractile **(A)** and [^3^H]-ACh release **(B)** effects in control conditions. The exposure of 1μM TTX and 100 nM mirabegron produced inhibition of both effects as shown in middle **(C, D)** and bottom **(E, F)** panels, respectively. In B, D and F diagrams, each point represents the radioactivity per gram of tissue in the superfusate.

EFS produced simultaneously a contractile response (IC_1_; 748.7 ± 20.8 mN, n = 15) and a marked ^3^ [H]-outflow (S_1_; 6,279 ± 798 Bq/g dry tissue, n = 15) (Figures 1A, B). In control experiments, the second EFS produced reduced effects on both patterns, showing an IC_2_/IC_1_ and S_2_/S_1_ ratio value of 0.90 ± 0.45 and 0.82 ± 0.02, respectively.

**FIGURE 2:**
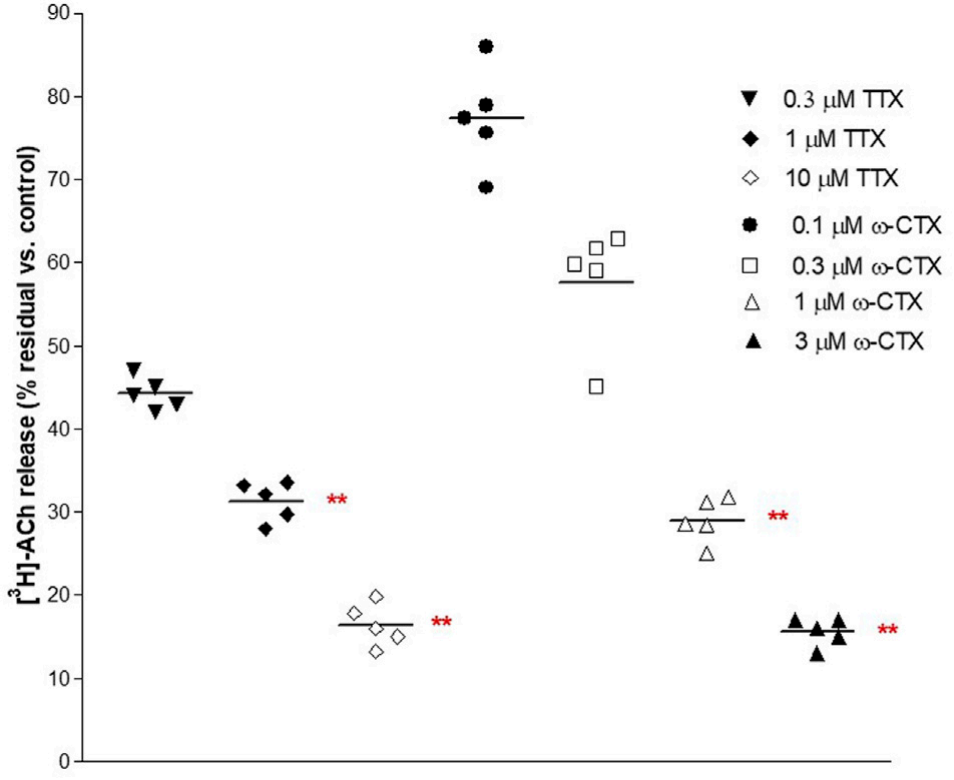
Concentration-dependent inhibition produced by 30 min exposure of TTX and ω−CTX on EFS-evoked [^3^H]-ACh release in pig DSM strips. Intermittent EFS (IS) was delivered at 20 Hz according to protocol A (see Methods for details). Data are the mean ± SD of n experiments. (*) *p* < 0.01; (**) *p* < 0.001 significance vs. controls.

These ratios were not significantly affected by the ganglionic blocker hexamethonium at 10 μM (n = 14, *p* > 0.05; not shown). Conversely, EFS-evoked contractile response and [^3^H]-outflow were reduced in a concentration-dependent manner by TTX assessed in 100 nM-10 μM concentration range (Emax at 10 μM: 92 ± 3% and 85 ± 2%, respectively; *n* = 5). A similar marked inhibitory pattern was observed for the N-type Ca^2+^-channel blocker ω-conotoxin GVIA (0.1–3 μM range) (Emax at 3 μM 70 ± 11% and 83 ± 5%, respectively; n = 5; Figure 2). Taken together, these data indicate that the [^3^H]-outflow mostly reflects the release of neural [^3^H]-ACh from cholinergic terminals, but it includes a TTX-insensitive component too (by about 15%–20%) likely related to the TTX-resistant spontaneous outflow (D'Agostino et al., 2015).

**FIGURE 3:**
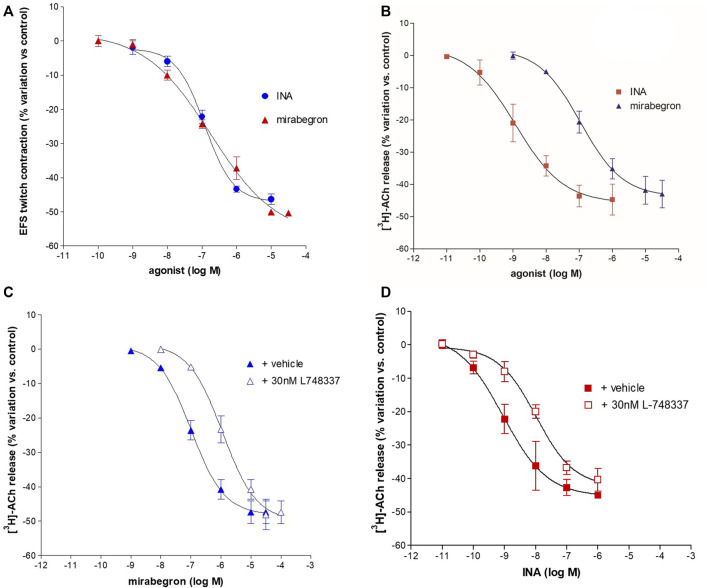
Concentration-dependent inhibition of INA and mirabegron on contraction **(A)** and [^3^H]-ACh release **(B)** evoked by intermittent EFS (IS) delivered at 20 Hz in pig DSM strips. In panel **(C, D)** the inhibitory effect on EFS-evoked-[^3^H]-ACh release in the presence of L748,337, selective antagonist for the β_3_-ADR subtype. Data are the mean ± SD of 5–14 experiments.

Isoprenaline (INA) and mirabegron (Figure 3A) inhibited the EFS-evoked contraction, producing CRCs with similar efficacy (Emax 50%) and potency (pEC_50_ 6.94 ± 0.07, *n* = 14, and 6.84 ± 0.29, *n* = 10, respectively).

EFS-evoked [^3^H]-ACh release was reduced in a concentration-dependent manner with similar Emax (45% and 40%, respectively; Figure 3B) but with a marked difference between the potencies of INA (pEC_50_ 9.06 ± 0.20, *n* = 14, and mirabegron (pEC_50_ 6.88 ± 0.19, *n* = 10). Notably, the same difference was also reported in the human detrusor (see Table 1) where an additional inhibitory β-ADR is present, resembling the β_2_-ADR subtype (see D’Agostino et al., 2015).

**TABLE 1:** Potency (pEC_50_), affinity (pK_i_) and variation values on EFS-evoked effects produced by ligands in pig DSM in comparison with respective values obtained in human DSM.

EFS-evoked effects	[^3^H] ACh release		Contraction	
Tissue	Pig DSM	Human DSM	Pig DSM	Human DSM
Parameter	pEC_50_/pK_i_/% variation		pEC_50_/pK_i_/% variation	
INA	9.06 ± 0.20	9.05 ± 0.18 (a)	6.94 ± 0.26	6.94 ± 0.07 (a)
Mirabegron	6.88 ± 0.19	6.89 ± 0.29 (a)	6.84 ± 0.29	6.91 ± 0.08 (a)
L748,337	8.56 ± 0.08	8.57 ± 0.12(a)	8.26 ± 0.18	8.10 ± 0.05 (f)
				
Paxilline	8.12 ± 0.95 facilitation		25.5 ± 1.64 facilitation	32 facilitation (b)
NS11021	13.96 ± 1.80 inhibition		12.14 ± 1.31 inhibition	
Apamine	33.39 ± 5.35 facilitation		33.64 ± 3.78 facilitation	32 facilitation (c, d)
SKA-31	32.65 ± 2.22 inhibition		31.63 ± 4.25 inhibition	33 inhibition (e)

a) Data from D’Agostino et al., 2015. Approximate values extrapolated from figures or diagrams reported from: Xin et al., 2012 (b); Afeli et al., 2012 (c); La Fuente et al., 2014 (d); Soder et al., 2013 (e); Propping et al., 2013 (f).

The [^3^H]-ACh release CRCs of mirabegron and INA were shifted to the right by the selective β_3_-ADR antagonist L-748,337 yielding a similar pK_i_ (8.56 ± 0.08, *n* = 10, and 8.56 ± 0.06, *n* = 14, respectively) (Figures 3C,D). The blocking action of 30 nM L-748,337 was estimated also on EFS-evoked contractile responses caused by mirabegron yielding a pK_i_ of 8.26 ± 0.18, *n* = 5, (Table 1). Taken together, both rank order of potencies and apparent affinities correlate with the presence of the β_3_-ADR subtype in porcine DSM (see Table 1).

### 3.2 Neural and muscular effects evoked by continuous EFS in pig DSM strips

**FIGURE 4:**
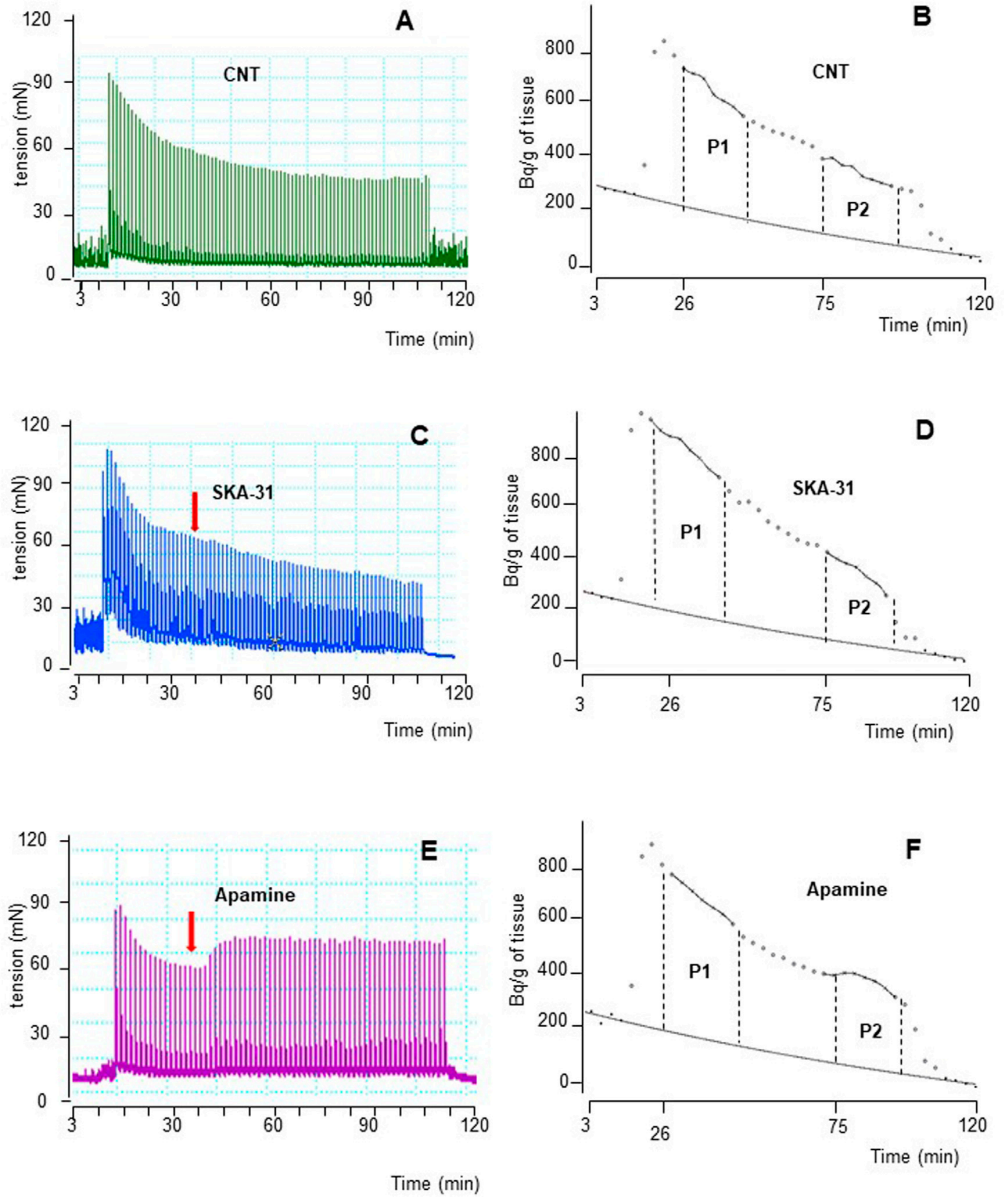
Time course illustrated by original drawings of experiments in pig DSM strips according to protocol B (see Methods for details). Contractile response and [^3^H]-ACh release were simultaneously evoked by EFS (CS) continuously delivered at 20 Hz, 60 s apart. Both effects are shown in control conditions **(A, B)**, in the presence of 10 μM SKA-31 **(C, D)** and of 100 nM apamine **(E, F)**. In B, D and F diagrams, each point represents the radioactivity per gram of tissue in the superfusate. *p* the amount of ACh released during 15 min period expressed as AUC (for details see Methods). Arrows indicate the starting time of drugs exposure.

The intracellular pathways suspected in the inhibitory effects caused by β_3_-ADRs activation were studied in a different set of experiments by means of subtype-preferring compounds for membrane K_Ca_ channels (BK and SK). For a reliable comparison, EFS was delivered with parameters of protocol B, previously used to stimulate human DSM strips (Soder et al., 2013). Continuous EFS caused DSM contraction (CC_1;_ 271.3 ± 65.1 mN, *n* = 7) and [^3^H]-ACh release (P_1_; 8,515 ± 1,126 Bq/g dry tissue, *n* = 7). A progressive reduction was observed during a period of 120 min showing a CC_2_/CC_1_ and P_2_/P_1_ ratio value of 0.79 ± 0.03 and 0.60 ± 0.02, respectively. The time course of an experiment in control conditions is shown in Figures 4A, B.

**FIGURE 5:**
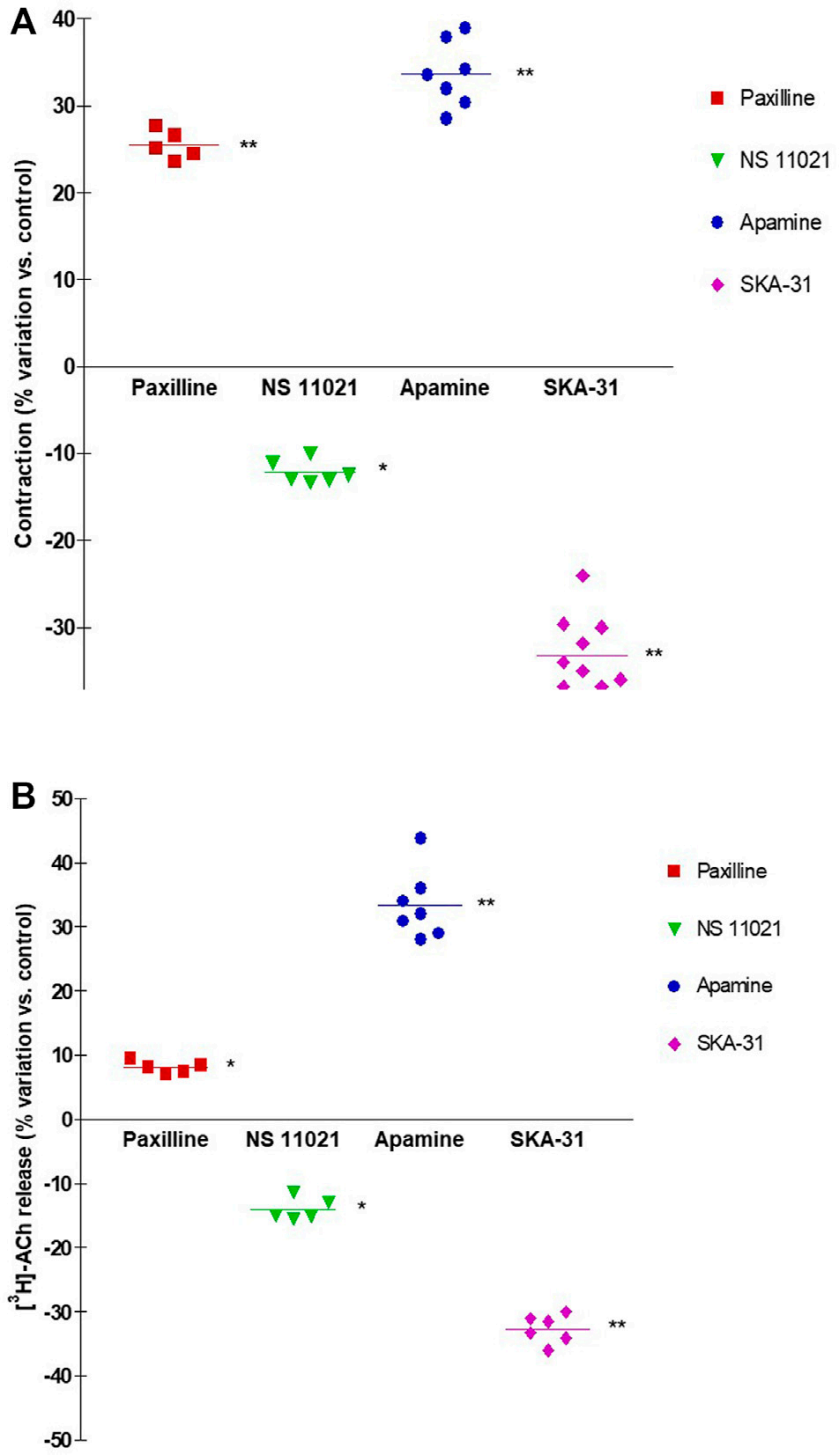
Comparison of DSM contraction **(A)** and neural [^3^H]-ACh release **(B)** produced by exposure of paxilline (1 μM), NS11021 (3 μM), apamine (100 nM) and SKA-31 (10 μM) in pig DSM strips. Contraction and [^3^H]-ACh release were simultaneously evoked by continuous EFS (CS) delivered with 3 s trains at 20 Hz, 60 s apart. Data are the mean ± SD of n experiments. (*) *p* < 0.01; (**) *p* < 0.001 significance vs. controls.

NS11021 (putative selective BK_Ca_ activator at 3 μM) caused small but significant reduction on contraction (8.75 ± 0.97%, *n* = 6) and [^3^H]-ACh release (9.2 ± 2.60%, *n* = 5) in six and five assays, respectively (*p* < 0.01). At variance, both effects were enhanced by 1 μM paxilline (BK_Ca_ blocker) (27.1 ± 2.5% and 8.12 ± 0.95%, respectively; *n* = 5, *p* < 0.01) (Figures 5A, B). Conversely, marked variation on DSM contraction was caused by the compounds selective for K^+^ channels of SK type, namely 10 μM SKA-31 and 100 nM apamin, which caused inhibition (32.44 ± 4.33%, *n* = 10) and facilitation (33.64 ± 3.78%, *n* = 7) of contractions, respectively (*p* < 0.001; Figures 4C,E; Figure 5A). Similarly, EFS-evoked [^3^H]-ACh release was reduced (32.65 ± 2.22%, *n* = 6) and enhanced (33.39 ± 5.35%, *n* = 7) by the SK ligands (*p* < 0.001; Figures 4D, F; Figure 5B). Noteworthy, the combination of the two drugs failed to affect both facilitation and inhibition significantly (not shown). The extent of variation on EFS-evoked contraction caused by the used compounds is listed in Table 1 for comparison with contractile experiments in human DSM.

## 4 Discussion

An increasing body of evidence supports the concept that the function of the human urinary bladder (during the storage and the voiding phase) depends on a complex interplay of several molecular pathways external to or within the bladder wall. (Andersson and Arner, 2004). In this respect, close similarities have been ascertained between human and porcine bladder in functional and mechanical properties (Parsons et al., 2012). In particular, similarities in receptors distribution (Goepel et al., 1997; Goepel et al., 1998) as well as pharmacological profiles of Ca^2+^ channels, involved in the contractile mechanism, have been described (Buckner et al., 2000; Kajioka et al., 2002; Hashitani and Brading, 2003). The excitatory component depends on ACh and ATP in both species (Kumar et al., 2004). Weather available evidence points to ACh as the main physiological neurotransmitter in humans (Andersson and Wein, 2004), the role of ATP rises in pathological conditions (Kumar et al., 2010) as well as in porcine detrusor when ATP breakdown is down-regulated (D’Agostino et al., 2012). In addition, the overall amount of released ATP, including the one released from neuronal (parasympathetic terminals) plus non-neural sources (urothelium) and ecto-ATPase activity affecting purine levels, is remarkably similar between pig and humans (Kumar et al., 2004). Moreover, the inhibitory sympathetic pathway involves β-ADR similar subtypes in both species (Yamaguchi, 2002; Yamanishi et al., 2002). In humans, β_3_-ADR located on DSM was considered the predominant subtype to cause effective bladder distension required for urine storage (Yamaguchi and Chapple, 2007). However, as recently focused (Okeke et al., 2017; Igawa et al., 2019), this concept has been recently more and more questioned because of the ambiguous reports on β_3_-ADRs localization (Coelho et al., 2017; Silva et al., 2017, 2020). Previous studies, based on validated antibodies (Limberg et al., 2010; Otsuka et al., 2013), showed that β_3_-ADRs are not only expressed on myocytes but also on other bladder wall structures suspected to contribute to the regulation of bladder function (Limberg et al., 2010).

The present functional experiments corroborate such assumption. Indeed, β_3_-ADRs activation by mirabegron caused smooth muscle relaxation simultaneously to a parallel reduction of ACh release from parasympathetic terminals. The concentration-dependent reduction of both effects was antagonized by the highly selective antagonist L-748,337, showing a pharmacodynamic pattern consistent with the involvement of the β_3_-ADR subtype at both sides (see Table 1 for comparison). Notably, in our porcine *in vitro* model, mirabegron is potent at a concentration therapeutically achieved in humans, a finding that does not corroborate classic pathophysiological and treatment concepts, historically focused on smooth muscle cells (Andersson, 2017). In addition, as previously suggested (D’Agostino et al., 2015), the observed reduction on the cholinergic motor drive might support the increase in post void residual volume documented in clinical trials (Drake et al., 2017), even if acute urinary retention remained negligible in OAB patients, where neurogenic detrusor contractions seem mostly purinergic in nature (D’Agostino et al., 2015).

It is evident that our findings stir up an open debate (Igawa et al., 2019), supporting the notion that the mechanism in reducing detrusor excitability might reside at neural site too. But, since OAB symptoms occur in the storage phase, it remains an enigma how neural ACh might contribute to an anomalous detrusor motor drive, since the parasympathetic motor drive is assumed to be “silent” during the filling phase (Igawa et al., 2019). Really, during the storage phase ACh (and ATP) are mainly released from urothelium, but urothelium does not represent the exclusive source. Indeed, in urothelium-deprived preparations parasympathetic nerves spontaneously release non-quantal ACh and detrusor stretch causes the increases non-neuronal ACh (Yoshida, et al., 2008). Both sources produce a valuable amount of ACh, claimed TTX-resistant. (D’Agostino et al., 1988; Zagorodnyuk, et al., 2009; Yoshida, et al., 2004). In the present study, our findings did not allow to establish whether β_3_-ADR agonists caused any reduction in the amount of ACh TTX-resistant (by about 15%–20% of the total amount evoked by EFS): this represents a not secondary matter that remains to be investigated in future specifically designed experiments.

As regards ATP, it was suggested that in human detrusors mirabegron could decrease the levels of neural ACh indirectly, promoting adenosine accumulation from myocytes into the neural cleft and, in turn, *via* activation of A_1_ receptors located at prejunctional site (Silva et al., 2017, 2020). However, it is reasonable to assume that such mechanism might work even more in neural cells, since higher concentrations of ATP (up to 100 mM) are stored in secretory vesicles of neurons and released with acetylcholine (and other transmitters) by nerve impulses (Zimmermann, 2008). On this subject, porcine detrusor might represent a useful tool to ascertain this molecular pathway in cholinergic terminals too, providing evidence to reinforce the neural hypothesis that β_3_-ADRs exert their inhibitory effect predominantly at pre-junctional site, even if by indirect mechanism underlying adenosine release generation. In this respect, isolated porcine detrusor was successfully used to explore the cross talk between purinergic and cholinergic pathways, a link of particular importance when the ATP breakdown is down regulated (D’Agostino et al., 2012). In fact, ATP and its metabolites influenced the ACh levels in the neural cleft of cholinergic terminals, determining an exaggerated cholinergic trend that, mimicking pathological situations in human bladders (Kumar et al., 2010; Silva-Ramos et al., 2020), resembled in porcine detrusor a condition of experimental hyperactivity.

Regarding the regulatory mechanism(s) underlying the above-described inhibitory effects, a recent study with adenylyl cyclase or PKA inhibitors showed that mirabegron-induced relaxation of pig and human detrusor smooth muscle occurs via both a β_3_-ADRs/cAMP-dependent and -independent pathway (Maki et al., 2019). Particularly in exocytosis, it is conceivable the involvement of biochemical cAMP-independent pathways regulating intracellular Ca^2+^ signals within the bladder wall. Indeed, nerve terminals contain a rich variety of cAMP/PKA-independent downstream effectors that ultimately govern neurotransmission. Among these, proteins as Ca^2+ −^activated K^+^ (K_Ca_) channels are prominent players in the control of neurotransmitters release (Wang, 2008). In the DSM cells, three major groups represent the K_Ca_ channel family: large conductance (BK_Ca_), intermediate conductance (IK_Ca_) and small conductance (SK_Ca_) channels (Petkov, 2011; Petkov, 2014). BK_Ca_ channels play an important inhibitory function in the bladder, in health as well as in LUTS condition (Hristov et al., 2011; Petkov, 2014). Notably, it has been exclusively ascribed to SK_Ca_ channels a negative feedback system controlling the neural activity in human DSM (Afeli et al., 2012; Hristov et al., 2012; Soder et al., 2013; La Fuente et al., 2014) as in pig DSM (Nielsen et al., 2011).

However, based on the suggestion that β-ADRs are functionally coupled to BK_Ca_ channels (Berkefeld et al., 2010) and that BK_Ca_ channels enrich a variety of presynaptic terminals regulating neurotransmitter release (Wang, 2008), we aimed to ascertain whether BK_Ca_ channels are really key negative regulators of cholinergic activity in the pig too.

The BK_Ca_ blocker paxilline caused a marked increase of DSM contractions (by about 25%) but not on neurotransmitter release (by about 8%). The enhancing effect of paxilline was counteracted by NS11021, a well-established highly specific activator for BK_Ca_ channels in 0.1–3 μM concentration range (Bentzen et al., 2014), necessarily chosen to avoid the drawbacks of non-selective BK_Ca_ openers (D’Agostino et al., 2017). The efficacy of NS11021 was evident at muscular side but not at neural level, even at the highest concentration (3 μM) (see Table 1). These data seem to exclude BK_Ca_ channels to exert a pivotal negative control at parasympathetic terminals, on the other hand a role not previously predictable in the absence of ACh release measurement. This finding might reinforce the notion that either the function of presynaptic BK channels may vary from synapse to synapse (Wang, 2008) or that efferent nerves in the bladder do not express functional BK_Ca_ channels (Werner et al., 2007).

In contrast, RT-PCR studies and immunohistochemistry analysis (Afeli et al., 2012) pointed out the occurrence of the SK3 channel as prominent subtype in whole detrusor tissue. Furtherly, the presence of SK3 channels is corroborated by functional data suggesting SK channels to exert a remarkable role in reducing nerve-evoked contractions in both human and pig detrusor (Nielsen et al., 2011; Soder et al., 2013). Our release experiments clearly demonstrate the involvement of SK3 channels in a negative feedback mechanism: indeed, compounds selective for the SK type, namely SKA-31 and apamin, markedly affected [^3^H]-ACh release and, in turn, DSM contractions (Figure 5). So it is now evident that SK channels, owing to their diverse subcellular and cellular expression, serve a diverse range of functions, from modulating repetitive firing patterns (Soder et al., 2013) to directly determining levels of neurotransmitter release. Thus, the modulation of intracellular second messenger pathways could offer promising possibility for selective relaxation of urinary bladder musculature. Noteworthy, the robust modulation produced by selective SK modulators on motor drive in pig, corroborating what reported in human DSM (see Table 1), might represent a novel strategy to develop new drugs for the treatment of human urinary bladder dysfunctions.

## 5 Conclusion

The present findings show close similarities between porcine and human detrusors regarding the functions of β_3_-ADRs in control the parasympathetic motor drive. Thus, this isolated animal model might represent a valid tool to study further mechanisms underlying the efficacy of β_3_-ADR agonists. In this respect, even if mirabegron has been defined as a β_3_-adrenoceptor agonist, off-target effects at other receptors and transporters have been reported (Dehvari et al., 2018) and worthy to be investigated.

In addition, experiments with porcine detrusor might be used translationally in pharmacology programs accompanying the clinical development of new β_3_-ADR agonists. In this respect, in pigs bred without estrogens we did not report any significant variation in the efficacy of mirabegron, at variance from the random efficacy observed in pigs of not selected strains used in a previous study (D’Agostino et al., 2017). The clinical meaning of our findings is yet to be determined. However, whenever β_3_-ADR agonists were used in female patients on estrogens therapy, our results might support a caveat for their clinical efficacy.

## Data Availability

The raw data supporting the conclusion of this article will be made available by the authors, without undue reservation.

## References

[B1] Afeli S. A. Rovner E. S. Petkov G. V. (2012). SK but not Ik channels regulate human detrusor smooth muscle spontaneous and nerve-evoked contractions. Am. J. Physiol. Cell Physiol. 303, F559–F568. 10.1152/ajprenal.00615.2011 PMC3423111 22592639

[B2] Andersson K. E. Arner A. (2004). Urinary bladder contraction and relaxation. Physiology and pathophysiology. Physiol. Rev. 84, 935–986. 10.1152/physrev.00038.2003 15269341

[B3] Andersson K. E. (2010). Detrusor myocyte activity and afferent signaling. Neurourol. Urodyn. 29, 97–106. 10.1002/nau.20784 20025035

[B4] Andersson K. E. (2015). Drug therapy of overactive bladder-what is coming next? Korean J. Urol. 56, 673–679. 10.4111/kju.2015.56.10.673 26495067 PMC4610893

[B5] Andersson K. E. (2017). On the site and mechanism of action of β3-adrenoceptor agonists in the bladder. Neurourol. J. 2, 6–11. 10.5213/inj.1734850.425 PMC5380826 28361520

[B6] Andersson K. E. Wein A. J. (2004). Pharmacology of the lower urinary tract: Basis for current and future treatments of urinary incontinence. Pharmacol. Rev. 56, 581–631. 10.1124/pr.56.4.4 15602011

[B7] Apostolidis A. (2015). Antimuscarinics in the treatment of OAB: Is there a first-line and a second-line choice? Curr. Drug Target 16, 1187–1197. 10.2174/1389450116666150518102021 25981605

[B8] Bentzen B. H. Olesen S. P. Rønn L. C. Grunnet M. (2014). BK channel activators and their therapeutic perspectives. Front. Physiol. 5, 389. 10.3389/fphys.2014.00389 25346695 PMC4191079

[B9] Berkefeld H. Fakler B. Schulte U. (2010). Ca2+-activated K+ channels: From protein complexes to function. Physiol. Rev. 90, 1437–1459. 10.1152/physrev.00049.2009 20959620

[B10] Buckner S. A. Milicic I. Daza A. Davis-Taber R. Scott V. E. Sullivan J. P. (2000). Pharmacological and molecular analysis of ATP-sensitive K (+) channels in the pig and human detrusor. Eur. J. Pharmacol. 400, 287–295. 10.1016/s0014-2999(00)00388-5 10988346

[B11] Coelho A. Antunes-Lopes T. Gillespie J. Cruz F. (2017). Beta-3 adrenergic receptor is expressed in acetylcholine-containing nerve fibers of the human urinary bladder: An immunohistochemical study. Neurourol. Urodyn. 36, 1972–1980. 10.1002/nau.23224 28185314

[B12] D'Agostino G. Bolognesi M. L. Lucchelli A. Vicini D. Balestra B. Spelta V. (2000). Prejunctional muscarinic inhibitory control of acetylcholine release in the human isolated detrusor: Involvement of the M4 receptor subtype. Br. J. Pharmacol. 129, 493–500. 10.1038/sj.bjp.0703080 10711347 PMC1571864

[B13] D'Agostino G. Chiari M. C. Grana E. (1988). Formation and release of [3H]-acetylcholine in the rat urinary bladder strip. J. Pharm. Pharmacol. 40, 7–9. 10.1111/j.2042-7158.1988.tb05140.x 2896783

[B14] D'Agostino G. Condino A. M. Calvi P. (2015). Involvement of β3-adrenoceptors in the inhibitory control of cholinergic activity in human bladder: Direct evidence by [3H]-acetylcholine release experiments in the isolated detrusor. Eur. J. Pharmacol. 758, 115–122. 10.1016/j.ejphar.2015.03.074 25861936

[B15] D'Agostino G. Condino A. M. Calvi V. Boschi F. Gioglio L. Barbieri A. (2012). Purinergic P2X3 heteroreceptors enhance parasympathetic motor drive in isolated porcine detrusor, a reliable model for development of P2X selective blockers for detrusor hyperactivity. Pharmacol. Res. 65, 129–136. 10.1016/j.phrs.2011.10.002 22041665

[B16] D’Agostino G. Condino A. M. Palea S. (2017). Selective β3-adrenoceptors agonists inhibit cholinergic activity in the urinary bladder: Direct evidence in isolate porcine detrusor by [3H]-acetylcholine release experiments. J. Neurochem. 142, 210. 10.111/jnc.13925

[B17] Dehvari N. da Silva Junior E. D. Bengtsson T. Hutchinson D. S. (2018). Mirabegron: Potential off target effects and uses beyond the bladder. Br. J. Pharmacol. 175, 4072–4082. 10.1111/bph.14121 29243229 PMC6177610

[B18] Drake M. J. MacDiarmid S. Al-Shukri S. Barkin J. Fianu-Jonasson A. Herschorn S. (2017). Adding mirabegron to solifenacin to treat overactive bladder has little impact on postvoid residual volume or urinary retention risk. Urology 104, 1–4. 10.1016/j.urology.2017.03.004 28322904

[B19] Goepel M. Gronewald A. Krege S. Michel M. C. (1998). Muscarinic receptor subtypes in porcine detrusor: Comparison with humans and regulation by bladder augmentation. Urol. Res. 26, 149–154. 10.1007/s002400050038 9631949

[B20] Goepel M. Wittmann A. Rùbben H. Michel M. C. (1997). Comparison of adrenoceptor subtype expression in porcine and human bladder and prostate. Urol. Res. 25, 199–206. 10.1007/BF00941983 9228673

[B21] Hanna-Mitchell A. T. Robinson D. Cardozo L. Everaer K. Petkov G. V. (2016). Do we need to know more about the effects of hormones on lower urinary tract dysfunction? ICI-RS 2014. Neurourol. Urodyn. 5, 299–303. 10.1002/nau.22809 PMC6103166 26872571

[B22] Hashitani H. Brading A. F. (2003). Electrical properties of detrusor smooth muscles from the pig and human urinary bladder. Br. J. Pharmacol. 140, 146–158. 10.1038/sj.bjp.0705319 12967944 PMC1573994

[B23] Haylen B. T. de Ridder D. Freeman R. M. Swift S. E. Berghmans B. Lee J. (2010). An International Urogynecological Association (IUGA)/International Continence Society (ICS) joint report on the terminology for female pelvic floor dysfunction. Neurourol. Urodyn. 29, 4–20. 10.1002/nau.20798 19941278

[B24] Hristov K. L. Chen M. Kellett W. F. Rovner E. S. Petkov G. V. (2011). Large-conductance voltage-and Ca2+-activated K+ channels regulate human detrusor smooth muscle function. Am. J. Physiol. Cell Physiol. 301, C903–C912. 10.1152/ajpcell.00495.2010 21697543 PMC3191561

[B25] Hristov K. L. Parajuli S. P. Soder R. P. Cheng Q. Rovner S. Petkov G. V. (2012). Suppression of human detrusor smooth muscle excitability and contractility via pharmacological activation of large conductance Ca2+-activated K+ channels. Am. J. Physio. Cell Physiol. 302, C1632–C1641. 10.1152/ajpcell.00417.2011 PMC3378020 22422396

[B26] Igawa Y. Aizawa N. Michel M. .C. (2019). β3 -Adrenoceptors in the normal and diseased urinary bladder-What are the open questions? Br. J. Pharmacol. 176, 2525–2538. 10.1111/bph.14658 30868554 PMC6592861

[B27] Kajioka S. Nakayama S. McMurray G. Abe K. Brading A. F. (2002). Ca2+ channel properties in smooth muscle cells of the urinary bladder from pig and human. Eur. J. Pharmacol. 443, 19–29. 10.1016/S0014-2999(02)01593-5 12044787

[B28] Kumar V. Chapple C. Chess-Williams R. (2004). Characteristics of adenosine triphosphate [corrected] release from porcine and human normal bladder. J. Urol. 172, 744–747. 10.1097/01.ju.0000131244.67160.f4abstract 15247774

[B29] Kumar V. Chapple C. R. Rosario D. Tophil P. R. Chess-Williams R. (2010). *In vitro* release of adenosine triphosphate from the urothelium of human bladders with detrusor overactivity, both neurogenic and idiopathic. Eur. Urol. 57, 1087–1092. 10.1016/j.eururo.2009.11.042 20022422

[B30] La Fuente J. M. Fernandez A. Cuevas P. González-Corrochano R. Chen M. X. Angulo J. (2014). Stimulation of large-conductance calcium-activated potassium channels inhibits neurogenic contraction of human bladder from patients with urinary symptoms and reverses acetic acid-induced bladder hyperactivity in rats. Eur. J. Pharmacol. 735, 68–76. 10.1016/j.ejphar.2014.03.060 24747752

[B31] Limberg B. J. Andersson K. E. Aura Kullmann F. Burmer G. deGroat W. C. Rosenbaum J. S. (2010). β-Adrenergic receptor subtype expression in myocyte and non-myocyte cells in human female bladder. Cell Tissue Res. 342, 295–306. 10.1007/s00441-010-1053-x 20953633 PMC3113530

[B32] Maki T. Kajioka S. Itsumi M. Kareman E. Lee K. Shiota M. (2019). Mirabegron induces relaxant effects via cAMP signaling-dependent and -independent pathways in detrusor smooth muscle. Low. Urin. Tract. Symptoms 11, O209–O217. 10.1111/luts.12247 30632283

[B33] Michel M. C. Korstanje C. (2016). β3-Adrenoceptor agonists for overactive bladder syndrome: Role of translational pharmacology in a repositioning clinical drug development project. Pharmacol. Ther. 159, 66–82. 10.1016/j.pharmthera.2016.01.007 26808167

[B34] Nielsen J. S. Rode F. Rahbek M. Andersson K. E. Rønn L. C. Bouchelouche K. (2011). Effect of the SK/Ik channel modulator 4,5-dichloro-1,3-diethyl-1,3-dihydro-benzoimidazol-2-one (NS4591) on contractile force in rat, pig and human detrusor smooth muscle. BJU In. 108, 771–777. 10.1111/j.1464-410X.2010.10019.x 21223472

[B35] Okeke K. Gravas S. Michel M. C. (2017). Do β3-adrenoceptor agonists cause urinary bladder smooth muscle relaxation by inhibiting acetylcholine release? Am. J. Physiol. Ren. Physiol. 313, F859–F861. 10.1152/ajprenal.00215.2017 28515177

[B36] Otsuka A. Kawasaki H. Matsumoto R. Shinbo H. Kurita Y. Iwashita T. (2013). Expression of β-adrenoceptor subtypes in urothelium, interstitial cells and detrusor of the human urinary bladder. Low. Urin. Tract. Symptoms. 5, 173–180. 10.1111/luts.12007 26663456

[B38] Parsons B. A. Drake M. J. Gammi A. Fry C. H. Vahabi B. (2012). The validation of a functional, isolated pig bladder model for physiological experimentation. Front. Pharmacol. 3, 52. 10.3389/fphar.2012.00052 22479248 PMC3315789

[B39] Patton D. M. (2021). Vibegron: A β3-adrenergic agonist for the treatment of overactive bladder. Drugs Today 57, 507–517. 10.1358/dot.2021.57.8.3293588 34405208

[B40] Petkov G. V. (2014). Central role of the BK channel in urinary bladder smooth muscle physiology and pathophysiology. Am. J. Physiol. Regul. Integr. Comp. Physiol. 307, R571–R584. 10.1152/ajpregu.00142.2014 24990859 PMC4166757

[B41] Petkov G. V. (2011). Role of potassium ion channels in detrusor smooth muscle function and dysfunction. Nat. Rev. Urol. 9, 30–40. 10.1038/nrurol.2011.194 22158596 PMC3759241

[B42] Propping S. Wuest M. Eichhorn B. Wirth M. P. Kaumann A. J. Ravens U. (2013). Mucosa of human detrusor impairs contraction and β-adrenoceptor-mediated relaxation. BJU Int. 112, 1215–1222. 10.1111/bju.12267 23937341

[B43] Silva I. Costa A. F. Moreira S. Ferreirinha F. Magalhães-Cardoso M. T. Calejo I. (2017). Inhibition of cholinergic neurotransmission by β3-adrenoceptors depends on adenosine release and A1-receptor activation in human and rat urinary bladders. Am. J. Physiol. Ren. Physiol. 313, F388–F403. 10.1152/ajprenal.00392.2016 28446460

[B44] Silva I. Magalhães-Cardoso M. T. Ferreirinha F. Moreira S. Costa A. F. Silva D. (2020). β3 Adrenoceptor-induced cholinergic inhibition in human and rat urinary bladders involves the exchange protein directly activated by cyclic AMP 1 favoring adenosine release. Br. J. Pharmacol. 177, 1589–1608. 10.1111/bph.14921 31721163 PMC7060368

[B45] Silva-Ramos M. Silva I. Faria M. Ferreirinha F. Correia-de-Sá P. (2020). Activation of prejunctional P2x2/3 heterotrimers by ATP enhances the cholinergic tone in obstructed human urinary bladders. J. Pharmacol. Exp. Ther. 372, 63–72. 10.1124/jpet.119.261610 31636173

[B46] Soder R. P. Parajuli S. P. Hristov K. L. Rovner E. S. Petkov G. V. (2013). SK channel-selective opening by SKA-31 induces hyperpolarization and decreases contractility in human urinary bladder smooth muscle. Am. J. Physiol. Regul. Integr. Comp. Physiol. 304, R155–R163. 10.1152/ajpregu.00363.2012 23174857 PMC3543661

[B47] Wang Z. (2008). Regulation of synaptic transmission by presynaptic CaMKII and BK channels. Mol. Neurobiol. 38, 153–166. 10.1007/s12035-008-8039-7 18759010 PMC2706205

[B48] Werner M. E. Knorn A. Meredith A. Aldrich R. Nelson M. (2007). Frequency encoding of cholinergic- and purinergic-mediated signaling to mouse urinary bladder smooth muscle: Modulation by BK channels. Am. J. Physiol. Regul. .Integr. Comp. Physiol. 292, R616–R624. 10.1152/ajpregu.00036.2006 16931654

[B49] Xin W. Cheng Q. Soder R. P. Rovner E. S. Petkov G. V. (2012). Constitutively active phosphodiesterase activity regulates urinary bladder smooth muscle function: Critical role of KCa1.1 channel. Am. J. Physiol. .Regul. Integr. Comp. Physiol. 303, F1300–F1306. 10.1152/ajprenal.00351.2012 PMC3518192 22896041

[B50] Yamaguchi O. (2002). Beta3-adrenoceptors in human detrusor muscle. Urology 59, 25–29. 10.1016/s0090-4295(01)01635-1 12007519

[B51] Yamaguchi O. Chapple C. R. (2007). Beta3-adrenoceptors in urinary bladder. Neurourol. Urodyn. 26, 752–756. 10.1002/nau.20420 17600372

[B52] Yamanishi T. Chapple C. R. Yasuda K. Yoshida K. Chess-Williams R. (2002). The role of beta3-adrenoceptors in mediating relaxation of porcine detrusor muscle. Br. J. Pharm. 135, 129–134. 10.1038/sj.bjp.0704470 PMC1573128 11786488

[B53] Yoshida M. Masunaga K. Satoj Y. Maeda Y. Nagata T. Inadome A. (2008). Basic and clinical aspects of non-neuronal acetylcholine: Expression of non-neuronal acetylcholine in urothelium and its clinical significance. Pharmacol. Sci. 106, 193–198. 10.1254/jphs.fm0070115 18285653

[B54] Yoshida M. Miyamae K. Iwashita H. Otani M. Inadom A. (2004). Management of detrusor dysfunction in the elderly: Changes in acetylcholine and adenosine triphosphate release during aging. Urology 63 (3), 17–23. 10.1016/j.urology.2003.11.003 15013648

[B55] Zagorodnyuk V. P. Gregory S. Costa M. Brookes S. J. Tramontana M. Giuliani S. (2009). Spontaneous release of acetylcholine from autonomic nerves in the bladder. Br. J. Pharmacol. 15, 607–619. 10.1111/j.1476-5381.2009.00166.x PMC2707973 19371347

[B56] Zimmermann H. (2008). ATP and acetylcholine, equal brethren. Neurochem. Int. 52, 634–648. 10.1016/j.neuint.2007.09.004 18029057

